# A cortical–hippocampal communication undergoes rebalancing after new learning

**DOI:** 10.1101/2025.03.26.645547

**Published:** 2025-03-29

**Authors:** Arron F Hall, Dong V Wang

**Affiliations:** 1 Department of Neurobiology & Anatomy, Drexel University College of Medicine, Philadelphia, PA 19129.

**Keywords:** CA1, ACC, CA1 deep and superficial sublayers, *in vivo* electrophysiology, sleep, memory, optogenetics

## Abstract

The brain’s ability to consolidate a wide range of memories while maintaining their distinctiveness across experiences remains poorly understood. Sharp-wave ripples, neural oscillations that occur predominantly within CA1 of the hippocampus during immobility and sleep, have been shown to play a critical role in the consolidation process. More recently, evidence has uncovered functional heterogeneity of pyramidal neurons within distinct sublayers of CA1 that display unique properties during ripples, potentially contributing to memory specificity. Despite this, it remains unclear exactly how ripples shift the activity of CA1 neuronal populations to accommodate the consolidation of specific memories and how sublayer differences manifest. Here, we studied interactions between the anterior cingulate cortex (ACC) and CA1 neurons during ripples and discovered a reorganization of their communication following learning. Notably, this reorganization appeared specifically for CA1 superficial (CA1sup) sublayer neurons. Utilizing a generalized linear model decoder, we demonstrate the pre-existence of ACC-to-CA1sup communication, which is suppressed during new learning and subsequent sleep suggesting that ACC activity may reallocate the contribution of CA1sup neurons during memory acquisition and consolidation. Further supporting this notion, we found that optogenetic stimulations of the ACC preferentially suppressed CA1sup interneurons while activating a unique subset of CA1 interneurons. Overall, these findings highlight a possible role of the ACC in rebalancing CA1 neuronal populations’ contribution to ripple contents surrounding learning.

## Introduction

Memories are not static, rather they are gradually consolidated into long-term traces across days and weeks (Klinzing et al., 2019; Squire et al., 2015). At any given moment, we are processing and consolidating an array of differing experiences. The ability to preserve distinctiveness across these various experiences is essential for memory consolidation. This requires a delicate balance between the mechanisms that facilitate the reactivation and transformation of new memories alongside those which regulate consolidation to prevent the intermingling of distinct experiences. While much attention has been focused on uncovering the principles that guide the reactivation and consolidation of memories, less is known about the mechanisms which regulate these processes and govern memory specificity.

During sleep, sharp-wave ripples (SWRs), neural oscillations predominantly within the CA1 of the hippocampus, facilitate hippocampal reactivations and accompany cortical reactivations of memory-related neurons, a process referred to as replay (Buzsáki, 2015; Buzsáki et al., 1992; Davidson et al., 2009; Diba & Buzsáki, 2007; Foster & Wilson, 2006; Lee & Wilson, 2002). Replay has been shown to be essential for memory consolidation as disruption or enhancement of ripples impairs or improves memory, respectively (Ego-Stengel & Wilson, 2010; Fernández-Ruiz et al., 2019; Girardeau et al., 2009; Gridchyn et al., 2020; Jadhav et al., 2012; Wang et al., 2015). Recently, evidence has uncovered functional and anatomical heterogeneity in CA1 neurons based on their position within the pyramidal layer: the superficial (CA1sup) and deep (CA1deep) sublayers (Danielson et al., 2016; Geiller et al., 2017; Hall & Wang, 2023; Harvey et al., 2023; Mizuseki et al., 2011; Sharif et al., 2021). CA1sup neurons have more stable firing rates and higher spatial acuity than CA1deep neurons (Danielson et al., 2016; Harvey et al., 2023; Mizuseki et al., 2011). Whereas CA1deep neurons respond more to enriched environments, sensory landmarks, and reward (Danielson et al., 2016; Geiller et al., 2017; Harvey et al., 2023; Sharif et al., 2021). During replays, CA1sup neurons show increased activity after spatial learning, whereas CA1deep neurons show decreased activity (Berndt et al., 2023; Harvey et al., 2023). Together, ripples and the recruitment of distinct subpopulations within CA1 drive new memory formation. Despite this understanding, the mechanisms which regulate their activity to balance consolidation across experiences remain less understood.

Emerging evidence has demonstrated the importance of suppression within the hippocampal network to balance neuronal excitability surrounding learning (Gava et al., 2024; Karaba et al., 2024; Norimoto et al., 2018). During pre-learning sleep, ripples depotentiate synapses which, if perturbed, impairs learning (Norimoto et al., 2018). Similarly, after learning, ripples facilitate the depotentiation of memory-unrelated neurons (Norimoto et al., 2018). Additionally, during post-learning sleep, hippocampal neurons initially display heightened firing rates before ultimately returning to baseline (Giri et al., 2019; Wilson & McNaughton, 1994). Prior studies have demonstrated that reestablishing baseline is necessary for learning, as excessive neuronal activity and synchrony impairs learning (Gava et al., 2024; Karaba et al., 2024). Suppression of the hippocampus, then, appears to play a key role in down regulating synapses, preventing memory saturation, and promoting memory flexibility (Balduzzi & Tononi, 2013). Interestingly, CA1sup neurons appear to be the most sensitive to this process (Gava et al., 2024). Following repetitive learning, CA1sup neurons become highly synchronized and coactive, which impairs future memory formation (Gava et al., 2024). Optogenetic inhibition of CA1sup, but not CA1deep neurons, after learning decouples and decreases neuronal activity which reduces rigidity and restores new memory formation (Gava et al., 2024). Still, exactly how facilitation and suppression are balanced throughout consolidation, and whether cortical inputs play a role in modifying hippocampal activity in this process, remains poorly understood.

Here, we employ *in vivo* electrophysiology recording across contextual fear conditioning (CFC) and sleep to understand how communications between the anterior cingulate cortex (ACC) and hippocampal CA1 are modified following learning. Previous studies have shown that the ACC displays increased activity immediately preceding CA1 ripples and is instrumental in CFC and memory consolidation (Einarsson & Nader, 2012; Frankland et al., 2004; Wang & Ikemoto, 2016). We uncovered a novel, sublayer specific, line of communication between ACC and CA1 surrounding ripples with ACC preferentially communicating with CA1sup neurons. This communication rebalances following learning, suggesting a potential role in memory consolidation.

## Results

### Pre-ripple ACC activity predicts CA1 activity during ripples

**Figure 1.**
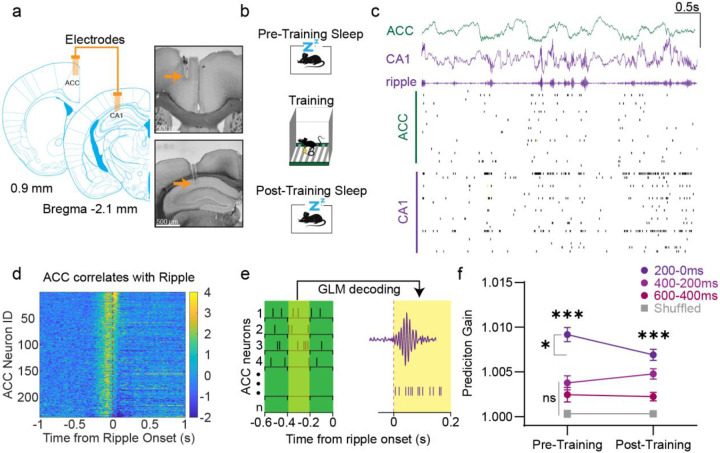
Pre-ripple ACC activity predicts CA1 activity during ripples. **a**, Left, schematic of a dual 8-tetrode array implanted in the ACC and CA1. Right, two representative brain sections highlighting the recording sites (orange arrows) in the ACC and CA1, top and bottom, respectively. **b,** Schematic of the contextual fear memory procedure. **c,** Top, Representative ACC and CA1 LFP from a pre-training sleep session, Y axis scale bar 1 mV. Bottom, individual spikes across ACC and CA1 neurons. **d,** Heatmap of ACC neuron (n = 238) activity during pre-training sleep surrounding ripple onsets (Bin = 5ms). **e,** Schematic of GLM decoder. 200ms bins of ACC spiking data preceding ripple predicts CA1 activity 0-100ms after ripple onset. **f,** Prediciton gain difference in decoding CA1 (n=217) activity between real versus shuffled ACC (n=238) activity. Mann Whitney-U two sided tests revealed significant differences between the real and shuffled data across all time windows (N = 8 animals), ***p <.001. Mann Whitney-U two sided tests revealed that prediction gain for the 200-0ms time window was significantly higher than 600-400ms and 400-200ms time windows in pre- (*p*<.001*)* and post-training sleep (*p*<.01). Wilcoxon signed rank test revealed significant differences between pre-training and post-training for 200-0ms window real data (p= .013) but not for the 600-400ms window (p= .845), 400-200ms window (p= .185), or the shuffled data (p=.524). Error bars indicate mean ± s.e.m. Note, there were no differences across any time window for the shuffled data (Kruskal-Wallis’ test, p=.145; supplemental figure 4). For clarity, the shuffled data presented here is the averaged shuffle data across all time windows.

To understand how ACC and hippocampal communication evolves surrounding learning, we employed simultaneous dual-site *in vivo* electrophysiology recordings of both regions throughout a contextual fear conditioning paradigm (Fig. 1 a&b). Local field potentials and neuronal spikes were recorded simultaneously, and slow-wave sleep was identified by CA1 delta waves and ripple oscillations (Wang et al., 2015) (Fig. 1c).

We first examined ACC neuronal spiking activity surrounding CA1 ripples during pre-training sleep. Across 8 mice, we recorded 238 ACC neurons, with individual counts per animal ranging between 21–47 neurons. Most ACC neurons (224/238) displayed significant changes in activity surrounding ripples compared to baseline (Fig. 1d; see Methods). Of the neurons with significant changes, the majority displayed a pre-ripple peak in activity (182/224; 81.25%) while only a minority (42/224 18.75%) showed a post-ripple peak in activity (Fig. 1d). To determine whether this correlated activity preceding ripples is indicative of information flow, we implemented general linear model (GLM) machine learning decoding to test whether ACC activity preceding ripples can predict CA1 activity during ripples (Fig. 1e) (Jun Liu et al., 2024; Rothschild et al., 2017). ACC spiking was binned into 200ms segments across multiple time windows to predict individual CA1 neuronal firing rates between 0-100ms after ripple onset (Fig. 1e; see Methods). The decoder was trained on 50% of the recorded data upon which the remaining 50% data was used for testing. All data collected for decoding was recorded during the first ~2 hours of pre- and post-training sleep. We found that across all time-windows, ACC prediction gain using real data was significantly higher than shuffled (Fig 1f). However, ACC prediction was greatest when ACC spiking was collected 200ms preceding ripple onset, which aligns with the observed correlated firing seen in Fig. 1d. In accordance, all subsequent GLM analyses in this paper will use the −200-0ms time window. We found that prediction gain for that time window was worsened after contextual fear conditioning, suggesting a learning-induced weakening in overall information flow from ACC to CA1 during ripples (Fig. 1f).

### Task-inactive CA1 neurons reshape their activity with ACC after learning

**Figure 2.**
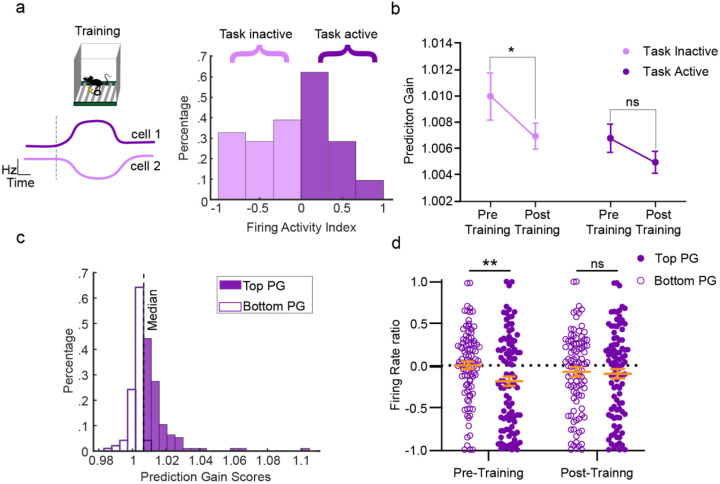
Task-inactive CA1 neurons reshape their activity with ACC after learning. **a**, Left, schematic of two represetnative neurons’ firing patterns during training, cell 1 increases (task-active) and cell 2 decreases (task-inactive) activity during training. Right, Frequency distribution histogram of firing activity index for CA1 neurons (n=190). 95 neurons display increased (task-active) activity, and 95 neurons display decreased (task-inactive) activity. **b,** Prediciton gain difference in ACC (N=207) decoding CA1 neurons based on whether they were task-active (N=95) or task-inactive (N=95). Error bars indicate mean ± s.e.m. Within subjects Wilcoxon signed rank test revealed significant differences between pre-training and post-training for task-inactive (*p*= .038) but not for task-active (*p*=.167). Mann Whitney-U two sided tests revealed no significant differences between task active/inactive neurons during pre- (p=.260) or post-training (*p*=.564) (N = 7 animals). **c,** Frequency distribution histogram of prediction gain scores for CA1 neurons (n=190; bin size .002). **d,** Firing activity index of CA1 neurons spilt based on their prediction gain score. Orange error bars indicate mean ± s.e.m. There was a significant difference in firing rate ratio between top 50% (N=95) versus bottom 50% (N=95) predictions scores for pre- (welch’s t-test *p*<.01) but not post-training (welch’s t-test *p*<.885).

Our GLM analysis provides a prediction gain score for each CA1 neuron, enabling us to examine how different properties of CA1 neurons may influence their prediction scores. We first aimed to investigate whether prediction gain scores across CA1 neurons differed based on their activity during training. To test this, we calculated a firing activity index ratio by comparing each CA1 neurons’ firing rate during training with its pre-training baseline (Fig. 2a) (Karaba et al., 2024). Neurons were separated into two categories based on whether they increased (task active) or decreased (task inactive) their activity during training (Fig. 2a). We found that, of the 190 CA1 neurons recorded, exactly one half increased while the other half decreased in activity during training (Fig. 2a). There were no pre-training baseline differences in firing rate between task active/inactive neurons (Supplemental Fig. 1a). We then asked whether prediction gain scores differed for task active versus task inactive CA1 neurons. Surprisingly, we found a significant learning induced decrease in prediction gain for task-inactive, but not task-active neurons, with no difference in prediction gain across groups (Figure 2b). These findings suggest that task-inactive CA1 neurons are suppressed from communication with the ACC during ripple-associated consolidation processes following learning (Fig. 2b).

Given that prediction gain scores are significantly higher during pre-learning sleep for task-inactive neurons, we asked whether the overall firing rate changes during learning differed based on the neurons’ prediction score strength. Therefore, we split CA1 neurons into two categories based on their prediction gain scores: the top 50% and bottom 50% scores during pre-training sleep (Fig. 2c). Our analysis showed that the CA1 neurons with the highest prediction scores (top 50%) had a significantly lower firing activity index compared to the neurons scoring in the bottom 50%. This suggests that neurons with greater pre-training predictive correlation with ACC are more likely to decrease their activity during learning (Fig. 2d). In contrast, CA1 neurons categorized based on post-training prediction gain scores do not show any difference in activity changes (Fig. 2d). The lack of difference in post-training sleep is notable, as it suggests that ACC heightened communication with task-inactive neurons is experience-dependent rather than intrinsic. These results further support our previous findings: the ACC is more strongly connected to CA1 task-inactive neurons during pre-learning sleep (Fig. 2d). Overall, these findings support the existence of pre-training ACC-to-CA1 communication, which is suppressed during new learning.

### ACC activity preferentially predicts CA1sup activity during ripples

**Figure 3.**
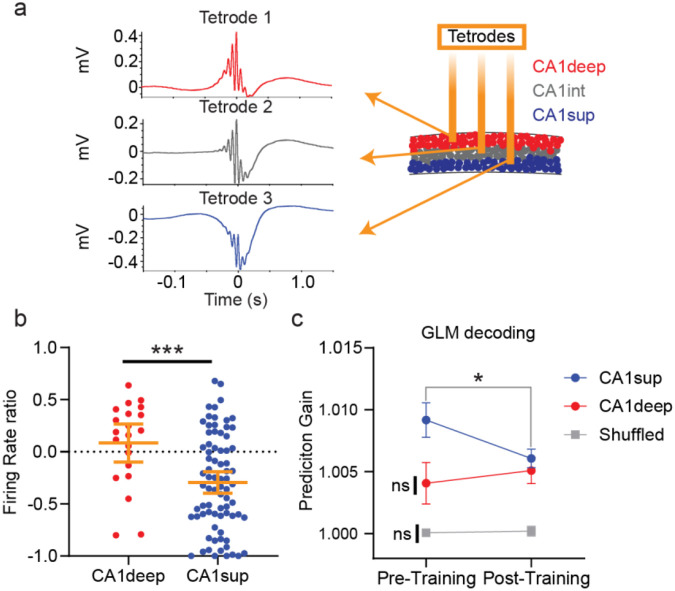
ACC activity preferentially predicts CA1sup activity during ripples. **a**, Schematic of Sublayer identification based on sharp-wave deflection difference across the pyramidal layer. **b,** Firing activity index comparison across sublayers during training, orange error bars indicate mean ± s.e.m. Mann-Whitney U revealed significant differences between CA1deep neurons (N=21; mean = .083) than CA1sup (N=77; mean. −.297), ***p<.001 (N=7 animals). **c,** GLM decoding of CA1 sublayers. Mann-Whitney-U two-sided test revealed significant differences between the real and shuffled data across pre- and post-training sleep for both sublayers), ***p <.001 (CA1deep N = 24; CA1sup N=94; N = 7 animals). There were no significant differences between sublayers during either pre- (*p*=.085) or post-training sleep (*p*=.915). Within subjects Wilcoxon signed rank test revealed significant differences between pre-training and post-training for CA1sup (*p*= .019) but not for CA1deep (*p*=.607) or shuffled (*p*=.988). Error bars indicate mean ± s.e.m.

It has been well established that functional heterogeneity exists within the pyramidal layer of CA1 (Hall & Wang, 2023; Mizuseki et al., 2011). To investigate whether ACC communication is weighted differently for CA1 sublayers, we separated CA1 neurons into CA1deep and CA1sup neurons based on their sharp-wave deflection characteristics (Fig 3a; for details see Methods) (Danielson et al., 2016; Mizuseki et al., 2011). We first examined the firing activity index during training and found sublayer differences with CA1deep neurons displaying significantly higher activity ratios than CA1sup (Fig. 3b). Overall, CA1deep tended to increase their activity during training (task active; mean = .083) while CA1sup neurons decreased their activity (task inactive; mean= −.297) (Fig 3b). In terms of GLM decoding (ACC→CA1), prediction gains based on real data were significantly higher than shuffled data for both CA1 sublayers. However, ACC showed a trend to more strongly predict CA1sup neurons (*p* =.083*)*, and they alone displayed learning induced changes. Together, this suggests that ACC preferentially communicates with CA1sup neurons, and that this communication is modified following learning (Fig 3c).

**Figure 4.**
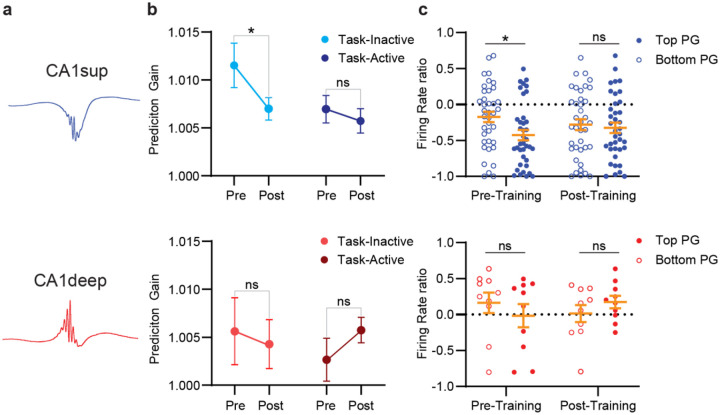
ACC activity preferentially predicts CA1sup task-inactive activity during ripples. **a**, Top, example waveform of CA1sup ripple. Bottom example waveform for CA1deep ripple. **b,** Prediciton gain difference in ACC (N=207) decoding CA1sup neurons based on whether they were task-active (N=95) or task-inactive (N=95). *Top,* Within subjects Wilcoxon signed rank test revealed significant differences between pre-training and post-training for CA1sup task-inactive (N= 53; *p*= .041) but not for CA1sup task-active (N=24; *p*=.568). Mann Whitney-U two sided tests revealed no significant differences between task active/inactive neurons at pre- (*p*=.141) or post-training (*p*=.509) sleep. *Bottom,* For CA1deep, there were no significant differences between pre- and post-training sleep for task-inactive (N=7; *p*=.300) or task-active (N= 14; *p*=.866) neurons. Mann Whitney-U two sided tests revealed no significant differences between task active/inactive neurons at pre- (*p*=.400) or post-training (*p*=.488) sleep. **c,** Firing activity index of CA1 sublayer neurons spilt based on their prediction gain score. *Top,* there was a significant difference in firing rate ratio between top 50% (N=38) versus bottom 50% (N=38) predictions scores of CA1sup neuon for pre- (t-test *p*=.016) but not post-training (t-test p=.669). *Bottom*. There were no differences in firing rate ratio between top 50% (N=38) versus bottom 50% (N=38) predictions scores of CA1deep for either pre- (t-test Mann-Whitney U *p*=.705) or post-training (t-test *p*=.247). Error bars indicate mean ± s.e.m.

Task-active and task-inactive neurons were present in both sublayers despite overall differences in firing activity index between layers. Therefore, we next examined whether prediction gain scores differed for task-active and task-inactive neurons within each sublayer. We found that CA1sup neurons showed significant decreases in prediction gain following learning for task-inactive but not task-active neurons (Fig. 4b). This difference was absent for CA1deep neurons (Fig. 4b), indicating sublayer functional differences. We next examined whether prediction gain strength was associated with firing rate changes during training across the sublayers. We found that CA1sup neurons with higher pre-training prediction gain scores displayed significantly decreased activity compared to CA1sup neurons with low prediction gain scores (Fig. 4c). Once more, there were no differences for CA1deep neurons (Fig. 4c). Overall, we find that ACC neurons preferentially communicate with CA1sup neurons during pre-learning sleep, and these CA1sup neurons are more likely to be task-inactive neurons. Learning appears to rebalance this communication, as predictability is weakened in post-training sleep.

### Optogenetic stimulation of the ACC preferentially inhibits CA1sup neurons

To determine whether the ACC can directly influence the activity of CA1sup neurons, we performed optogenetic stimulation of ACC neurons during sleep while recording CA1 neurons. We unilaterally microinjected AAV-CaMKII-ChR2 into the ACC and ipsilaterally implanted an optic fiber above the injection site, alongside a recording tetrode array in CA1 (Fig 4a; Supplemental Fig. 5). After waiting for viral expression, we administered 4 pulse 25Hz optogenetics stimulations during sleep to examine how CA1 neurons responded to ACC stimulations. CA1deep and CA1sup neurons both showed changes upon stimulations, albeit to different capacities (Fig. 4b-c). CA1sup neurons displayed prolonged activity changes, primarily suppression, whereas CA1deep showed weaker transient responses. To quantify CA1sublayer differences, we normalized CA1 firing rates across the stimulation window (0 to 4s from stimulation onset) and compared pre-stim to post-stim normalized firing rate (Fig 4d). We found that CA1sup firing rate was significantly lower than CA1deep neurons during the 4s post-stim window. Investigating further, we found that differences between sublayers lasted up to 3 seconds after stimulation, highlighting a prolonged impact of ACC stimulations on CA1sup neuronal activity (Fig 4e). Of note, we replicated these stimulation parameters during wakefulness and found more muted responses across each sublayer, suggesting communication between regions is greater during sleep (Supplemental Fig. 2). Overall, activating ACC produces long-lasting inhibition of CA1sup with minimal impact over CA1deep neurons, supporting a specific ACC-CA1sup line of communication during sleep.

### Optogenetic stimulation of the ACC differentially affects CA1 interneurons.

**Figure 5.**
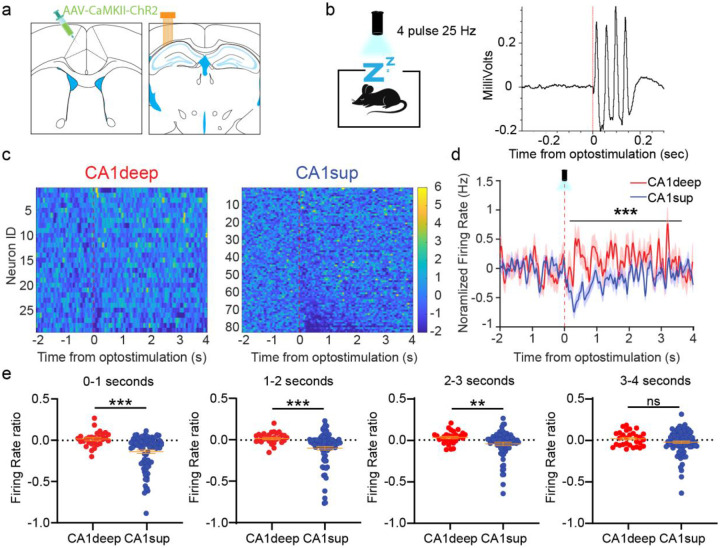
Optogenetic stimulation of the ACC preferentially inhibits CA1sup neurons. **a**, Schematic of optic fiber and microdive implant. **b,** Left, schematic of optogenetic manipulation. Four pulse 25Hz stimulations were performed during home-cage sleep. **c,** Right, representative CA1 LFP response to ACC stimulations. CA1 displays a maximal inhibitory peak roughly 13ms after stimulation. **d,** Heatmap activity CA1deep (N=29) and CA1sup neurons (N=84). **e,** Averaged CA1deep (red) and CA1sup (blue) normalized firing rate across −2-4s surrounding ACC stimulation. Independent samples t-test revealed significant differences between CA1deep and CA1sup activity in the post-stimulation window **p<.001, shaded region error bars indicate mean ± s.e.m. **f, F**iring rate ratio comparison between CA1deep and CA1sup neurons post-stimulation. Mann-Whintey U test revealed significant differences between groups across the first 3 seconds after stimulation, ***p <.001, **p.< 01. However, there was no difference 3-4s after stimulation, *P*=.324. Orange error bars indicate mean ± s.e.m.

**Figure 6.**
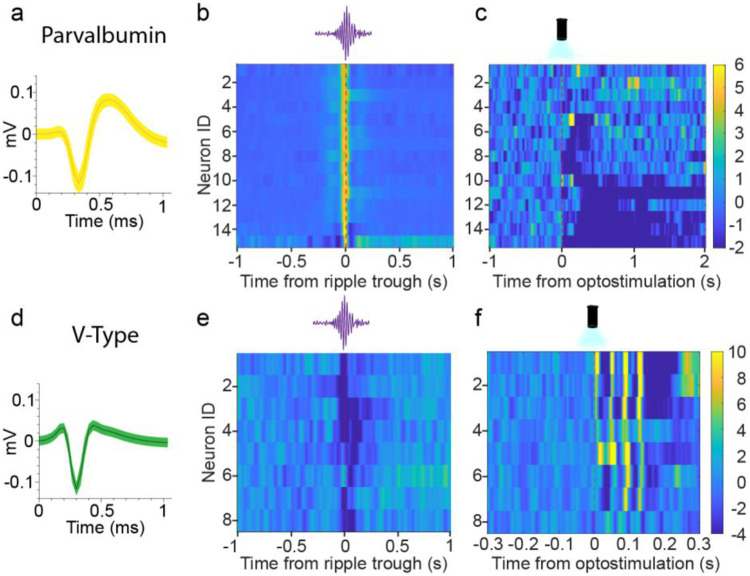
Optogenetic stimulations of the ACC differentially affect CA1 interneurons. **a**, Representative waveform of putative parvalbumin interneuron. Heatmap of PV interneurons spiking activity during ripples (bin size, 10ms; N=15). **c,** Heatmap of PV interneurons spiking activity following stimulation of the ACC (bin size, 10ms; N=15). **d,** Representative waveform of a V-type interneuron. **e,** Heatmap of V-type neurons interneurons spiking activity during ripples (bin size, 10ms; N=8). **f,** Representative heatmap of V-type interneurons spiking activity following stimulation of the ACC (bin size, 3ms; N=8).

Within CA1 lies a dense and complex circuit of interneurons, which play critical roles in memory processes (Tzilivaki et al., 2023). We aimed to understand how local interneurons within CA1 respond to ACC stimulations. We first investigated the responses of fast-spiking putative parvalbumin neurons (PV) (Fig 6a). PV firing properties have been well characterized and have been shown to display increased activity during ripples (Fig 6b; see Methods for details) (Klausberger & Somogyi, 2008; Opalka et al., 2020; Varga et al., 2014). Upon stimulation of the ACC during sleep, we discovered a robust inhibition of PV interneurons (Fig. 6c). Like CA1sup neurons, PV suppression was delayed (mean latency =.13s, SD = .15s) and prolonged, demonstrating that the ACC can drive the sustained suppression of both CA1sup and PV interneurons (Fig. 5c; Fig. 6c).

Our CA1 LFPs exhibited a laser-evoked response peaking roughly 13ms after ACC stimulation, which precedes many of the responses seen in CA1sup (mean latency .97s, SD = 1.04s) and PV interneurons (mean latency =.13s, SD = .15s) (Fig. 5b&c; Fig. 6c). This suggests the presence of a different population of CA1 neurons that respond on a scale similar to that seen in the CA1 LFP. Throughout our recording sessions, we noticed a reoccurring neuron type that showed a distinct V-shape waveform (Fig 6d). Notably, these V-type interneurons displayed distinct activity patterns during sleep, i.e., decreasing their activity during ripples. Unlike pyramidal cells and PV interneurons, which showed delayed and prolonged inhibition, V-type neurons displayed low latency (mean latency = 17ms, SD = 22ms) and brief excitatory responses to the stimulations (Fig. 5f). Given that V-type neuron activation precedes suppression pf pyramidal cells and PV interneurons after ACC stimulation, it is possible that V-type neurons mediate the inhibition seen in pyramidal cells and PV interneurons. However, further studies are needed to identify and test whether these neurons influence CA1 network activity.

## Discussion

Balancing network excitability with suppression after learning has long been thought to be essential for memory formation, with sleep serving a key homeostatic role (Liu et al., 2010; Tononi & Cirelli, 2003; Tononi & Cirelli, 2006; Watson et al., 2016) Excessive excitability and overly strengthened synapses can hinder neuronal flexibility and saturate the capacity for learning (Balduzzi & Tononi, 2013; Gava et al., 2024; Norimoto et al., 2018). Additionally, underregulated excitability may pathologically link multiple memory traces, leading to memory overgeneralization (Jinde et al., 2012; Kim et al., 2021). Therefore, regulatory mechanisms that rebalance the excitability of neurons to enable neuronal flexibility are essential for memory formation (Gava et al., 2024; Karaba et al., 2024). CA1sup neurons have been shown to be particularly susceptible to memory rigidity (Gava et al., 2024; Hall & Wang, 2023). While activation of these neurons is necessary for the encoding of new memories, their regulation and timely suppression appear equally important to evoke flexibility and enable *de novo* consolidation within the hippocampal network (Gava et al., 2024; Harvey et al., 2023). Our results reveal a rebalancing of communication between the ACC and hippocampus following learning. Specifically, we find that ACC displays a trend for stronger communication, as determined by prediction gain, with CA1sup neurons during pre-learning sleep. CA1sup neurons with higher prediction gains were more likely to display decreased activity during training. After learning, this communication undergoes reconfiguration. Prediction gain of CA1sup neurons is weakened and ACC shows no sublayer preference in communication. Overall, our study uncovers an ACC-CA1sup line of communication that adapts to learning, potentially playing a role in the rebalancing of network excitability for memory formation.

### Pre-ripple ACC activity is associated with CA1sup task-inactive neurons.

We implemented generalized linear models to decode CA1 neuronal firing rates during ripples based off the spiking rates of large ACC neuronal populations preceding ripples (Rothschild et al., 2017). The underlying principles of this analysis posit that if ACC activity preceding ripples influences subsequent activity in CA1 during ripples, the specific activity pattern of ACC neurons (predictor cells) should hold relevancy over the outcome of CA1 neurons (predicted cells). We found this to be true across all sleep sessions and neuron types, with real ACC spiking data showing significantly higher prediction gain compared to shuffled data, indicating an information flow from ACC to CA1 surrounding ripples. Interestingly, prediction gain was weakened following learning specifically for task-inactive CA1sup neurons. This suggests that ACC activity during pre-training sleep may predispose CA1sup neurons to be less active during subsequent training. However, questions remain over exactly how the ACC influences CA1sup activity and why this information flow rebalances after learning. Below we highlight two possible explanations.

One possible explanation is that ACC acts to reduce CA1sup excitability to prevent rigidity by decreasing their likelihood for involvement in upcoming encoding/consolidation. We found that ACC stimulation preferentially inhibits CA1sup neurons during sleep. These stimulations were prolonged, on the order of seconds, demonstrating that ACC can induce long-lasting suppression in CA1sup neurons. It is possible, then, that our observed communication flow between ACC-CA1sup is mainly inhibitory and that reducing CA1sup neurons makes them less likely to be involved in subsequent encoding (Guskjolen & Cembrowski, 2023). Accordingly, we found that CA1sup neurons with greater functional connectivity are more likely to be task-inactive. Therefore, the ACC may act to suppress CA1sup neurons to either promote memory flexibility or enhance the signal-to-noise ratio of information coding for other CA1 neurons, including CA1deep neurons (Balduzzi & Tononi, 2013; Gava et al., 2024). Nevertheless, future tests examining how ACC stimulation impacts learning and CA1sup activity are needed to parse out these details.

Another possible explanation is that ACC-CA1sup communication before training may reflect encoding of past experiences. Research has demonstrated that the ACC has a limited role in consolidation immediately after learning (Junyu Liu et al., 2024). Instead, memories become ACC-dependent beginning typically two days after learning (Einarsson & Nader, 2012; Frankland et al., 2004; Junyu Liu et al., 2024). It is possible that enhanced pre-learning communication between ACC-CA1sup is reflective of consolidating past experiences. If true, decreased activity of ACC associated CA1sup neurons may be due to the network’s shift towards prioritizing the encoding/consolidating of the new experience and thus the role of the ACC to CA1sup, consolidating past memories, is reduced (Diekelmann & Born, 2010; Huelin Gorriz et al., 2023; Wilson & McNaughton, 1994). Future experiments casually manipulating this communication are needed to determine whether ACC activity directly influences the downregulation of CA1sup activity during training, or whether these regions are correlated partners whose activity is ultimately mediated by an external brain region.

As research investigating CA1 heterogeneity continues to gain attention, functional distinctions across different behaviors have emerged (Hall & Wang, 2023). Previous research has found that CA1deep neurons are more involved in reward and sensory landmark encoding, while CA1sup neurons are more involved in context and spatial encoding (Danielson et al., 2016; Geiller et al., 2017; Harvey et al., 2023). Likewise, CA1deep place cells are more likely to remap in response to local cue changes than CA1sup place cells (Esparza et al.). Here, we add to this understanding with CA1sup neurons having a diminished role in fear memory formation. There have been conflicting reports over which sublayer is more involved in learning and replay (Berndt et al., 2023; Geiller et al., 2017; Hall & Wang, 2023; Harvey et al., 2023). We speculate that it may depend on the behavioral task. Past reports which have shown that CA1sup neurons are more involved in memory replay, implemented spatial learning tasks (Berndt et al., 2023; Harvey et al., 2023). In contrast, CA1deep neurons have been shown to be more important for encoding reward (Danielson et al., 2016; Harvey et al., 2023). Here, we found a trend for CA1deep neurons increasing their activity during contextual fear training, suggesting a potential role in encoding fear memories. Therefore, encoding and replay of more episodic-like features such as reward or fear learning may primarily be mediated by CA1deep neurons whereas spatial memories may be facilitated by CA1sup. Yet, recent findings complicate this notion, demonstrating sublayer differences emerging between two classes of ripples that differ based on current source density profiles (Castelli et al., 2025). Ultimately, future studies are needed to fully dissect the functional differences among CA1 sublayers during ripples and across behaviors.

### Optogenetic stimulation of the ACC inhibits CA1sup neurons and PV interneurons.

We found that cell body stimulation of ACC excitatory neurons inhibited CA1sup and PV interneurons. Notably, these responses were delayed for CA1sup and PV, indicating polysynaptic connectivity (Piñol et al., 2012). Additionally, both neuron types exhibited sustained suppression in response to stimulations suggesting that the ACC may have an indirect and broad influence over CA1 network activity rather than a direct and transient role. Sustained inhibition over certain populations of pyramidal neurons and PV interneurons may support a rebalancing of network excitability, especially during sleep (Diekelmann & Born, 2010; Norimoto et al., 2018; Tononi & Cirelli, 2003).

The identification of V-type neurons may help explain responses seen in CA1sup and PV interneurons. These neurons displayed the lowest latency responses to stimulations and produced brief excitatory bursts indicative of a more direct response to ACC stimulations (Piñol et al., 2012). These neurons share features with CCK expressing interneurons, such as decreased activity during ripples (Dudok et al., 2021; Vancura et al., 2023). It is possible that V-type neurons may overlap with CCK interneurons. Should this be verified, CCK interneuron functionality may explain some of the phenomena we see with the optogenetics findings. Namely, CCK interneurons are known to target CA1sup and PV interneurons and induce long-term excitability changes as seen in our optogenetic experiments (Hefft & Jonas, 2005; Karson et al., 2009; Klausberger et al., 2005; Valero et al., 2015). More recently, CCK interneurons have been identified as playing a causal role in regulating the excitability of CA1 neurons during ripples (Karaba et al., 2024). Together, this suggests a possible ACC influence on CA1 CCK neurons, which provides sustained inhibition of CA1 pyramidal and PV interneurons during sleep in facilitating memory formation. However, these interpretations remain speculative as further studies are needed to characterize V-type neurons, and casual experiments are required to completely elucidate their role. Finally, an important caveat to mention is that it remains an ongoing debate whether ACC directly projects to CA1 (Andrianova et al., 2023; Rajasethupathy et al., 2015; Shi et al., 2022). While our LFP and V-type responses to ACC stimulation have a shortened latency ~13-17ms, future anatomical studies are needed to conclusively determine connectivity (Cho et al., 2013; Petreanu et al., 2007; Wang et al., 2009).

## Methods

### Mice.

Male C57BL/6 mice were purchased from the Jackson Laboratory (stock #000664). Mice were 3–4 months old at the time of surgery; after surgery, they were singly housed in cages (40 × 20 × 25 cm) containing corn cob and cotton material and kept on a 12 h light/dark cycle with *ad libitum* access to food and water. Experimental procedures were approved by the Institutional Animal Care and Use Committees of Drexel University (protocol # LA-23-740) and were in accordance with the National Research Council *Guide for the Care and Use of Laboratory Animals*.

### Stereotaxic surgery.

Surgery procedures were similar to that used in our lab (Huang et al., 2025; Jun Liu et al., 2024; Liu et al., 2021). In brief, mice were anesthetized with ketamine/xylazine mixture (~100/10 mg/kg, i.p.) and kept on a heating pad at 37°C. For *in vivo* electrophysiology recording, mice received implantation of two electrode arrays (8 tetrodes each) into the ACC and CA1, respectively (Lin et al., 2006). For optogenetic stimulation, mice received intra-ACC microinjection of AAV viruses (AAV1-CKIIa-ChR2-GFP; 0.250 μl; ~10^13^ GC/ml; *Addgene* 105669) and implantation of one optic fiber (diameter 200 μm) slightly above the injection site; meanwhile, they received implantation of 8 tetrodes into the CA1 unilaterally on the ipsilateral side. AAV viruses were microinjected through a syringe pump (*World Precision Instruments*) over 5 min (50 nL/min), with an additional 5 min waiting period before removal of the injection needle (34 gauge, beveled). ACC coordinates from Bregma were AP 0.9 mm, ML 0.3 mm, DV 1.0 mm; CA1 coordinates from Bregma were AP −2.1 mm, ML 1.7 mm, and DV 1.1 mm.

### *In vivo* electrophysiology.

Each tetrode consisted of four wires (90% platinum 10% iridium; 18 μm diameter; *California Fine Wire*). A microdrive was used to couple with the electrode array, similar to that used in our lab (Jun Liu et al., 2024; Liu et al., 2021; Wang et al., 2015). Neural signal was preamplified, digitized, and recorded using a *Blackrock Neurotech* CerePlex. The local field potentials (LFPs) were digitized at 2 kHz and filtered at 500Hz low cut; spikes were digitized at 30 kHz and filtered between 600–6000 Hz. The tetrode arrays were gradually lowered daily until we recorded clear ripples and a substantial number of neurons; otherwise, mice were excluded from the study. The recorded spikes were sorted manually using *Plexon* Offline Sorter. For dual-site experiments, spikes from 8 mice were used for analyses in this study; the neuron numbers in ACC and CA1 were 47/27, 35/51, 28/12, 32/27*, 22/29, 26/21, 28/33, and 21/17 respectively. For optogenetic and *in* vivo electrophysiology experiments, 5 mice were used. The neurons numbers in CA1 were: 35, 30, 10, 38, and 24.

* Animal missing recording file during training and therefore was excluded from analyses comparing task active/inactive and firing rate ratios during training.

### Optogenetics manipulations:

Following surgery, mice were given 1 week for recovery and viral expression development. During recording, mice received 4pulse 25Hz stimulations (20s intertrial interval). Laser stimulations ranged between 5mW-8mW (473 nm wavelength). Laser intensity was tuned for each recording session until stimulation evoked a >0.2mV change in CA1 LFP.

### Ripples.

Ripples were band-pass filtered between 100–250 Hz and ripple envelope was smoothed with a Gaussian kernel (s.d. = 4 ms) (Karlsson & Frank, 2009). Ripple amplitudes were defined as the peak values of ripple envelopes. For analyses, we used amplitudes exceeding 5 s.d. above the mean. Ripple onsets and offsets were defined as the points where ripple amplitudes exceeded 1 s.d. above the mean before and after the corresponding ripple peaks (Fig. 3a). Ripple length was defined as the duration between the onset and offset; only ripples longer than 20 ms were used for further analysis.

### Task-active and task-inactive neurons.

Task active and inactive neurons were defined based on whether their training firing rate ratio over baseline (calculated in pre-training sleep) was positive (task-active) or negative (task-inactive) (Girardeau et al., 2017; Karaba et al., 2024; Oliva et al., 2020). Equation: (THz − PreHZ) / (THz + PreHz) in which THz and PreHZ are the average spikes per second of each CA1 neurons calculated during training and pre-training sleep, respectively.

### GLM decoding.

We constructed generalized linear models (GLMs) with a log link function to predict spike counts of individual CA1 neurons during ripples based on population spike counts in ACC across specific time windows (Jun Liu et al., 2024; Rothschild et al., 2017). Spike counts of each neuron were binned in 200-ms bins relative to ripple onset: −600 to −400 ms, −400 to −200 ms, −200 to 0 ms to predict CA1 activity 0–100ms. We randomly partitioned the ripples into two equally sized datasets: one of them was used to train the GLM decoder, and the other was used for testing. The model derived from the training phase was applied to the ACC population spike data in the test set, yielding predictions for the predicted CA1 spike counts across ripples. We calculated a prediction error for each CA1 neuron that was the mean difference between the predicted spike rate and real spike rate. We replicated this same analysis for shuffled data. Prediciton gains were derived by dividing shuffled prediction error by the real prediction error.

### ACC activity surrounding ripples.

To calculate significant differences in ACC activity surrounding ripples, we computed peri-ripple event histograms (smoothed with a three-bin Gaussian filter; bin size, 5 ms) for individual ACC neurons. We then compared ACC spiking activity between two 250ms windows starting either 200ms or 2000ms (baseline) before ripple onset. 224 neurons (94%) showed significant differences from baseline (Wilcoxen-signed rank *p*<.01*)*. Pre- or post-ripple peaks were determined whether a neurons firing rate peaked before (200 to 0ms) or after (0-200ms) ripple onset.

### Response latency to ACC optogenetic stimulations.

Neural activity following stimulation onset was binned (CA1sup 20ms; PV 10ms; V-type 3ms) and z scored. Response latency was obtained by selecting the first bin where a neuron showed a difference from the baseline by a z-score of ± 2 or more (minimum for 3 consecutive bins).

### Classification of CA1 pyramidal and interneurons.

Putative excitatory (pyramidal) neurons and parvalbumin fast-spiking interneurons were classified based on spike waveform, firing rate, and spike width (Opalka et al., 2020) (Supplemental Fig. 3). V-type neurons were classified based on waveform, firing rate, spike width, and decreased ripple related activity (Supplemental Fig. 3). For sublayer classification, we examined the mean amplitude of the sharp wave deflection at maximum ripple power for each tetrode. Sharp-wave deflection means that were greater than 50μV were classified as targeting the deep sublayer whereas tetrodes with a deflection less than −50 μV were classified as superficial layer tetrodes. Any deflection which had peaks that spanned both positive and negative voltage range, were treated as intermediate and were excluded from sublayer analyses (Figure 3a).

### Fear conditioning (*in vivo* recording).

The fear-conditioning chamber used in the experiment was a square chamber measuring 25 × 25 × 32 cm, with a 36-bar shock grid floor (*Med Associates*). The behaviors of the mice were recorded using *Blackrock Neurotech NeuroMotive* video system. During training, the mice were first allowed to explore the footshock chamber for 3 minutes. They then received 3 mild footshocks (0.75 mA, 0.5 s), with a 2min interval between shocks. About 30 seconds after the last shock, the mice returned to their home cages. After ~2 hours of post-training sleep, the mice were placed back in the footshock chamber for a 5-min contextual fear test. Neural activity was recorded continuously, including the pre-training sleep (2-3 hours), training (7.5 min), post-training sleep (2-3 hours), and contextual-fear test (5 min). Additionally, the day prior to fear conditioning, mice were placed into a novel box for 7.5min; neural activity was recorded across the pre-exposure sleep, exposure, and post-exposure sleep phases.

### Histology.

To mark the final recording sites, we made electrical lesions by passing 20-second, 10-μA currents through two or more tetrodes. Mice were deeply anesthetized and intracardially perfused with ice-cold PBS or saline, followed by 10% formalin. The brains were removed and postfixed in formalin for at least 24 hours. The brains were sliced into coronal sections of 50-μm thickness using *Leica* vibratome. Sections were mounted with Mowiol mounting medium mixed with DAPI for microscopic examination of electrode placements, viral vector expression, and/or optical fiber placements.

### Statistics.

Sample sizes were based on previous similar studies in our labs (Jun Liu et al., 2024; Liu et al., 2021; Wang et al., 2015). Other statistical analyses include Freidman’s test, Wilcoxon signed-rank test, Mann-Whitney U test, Kruskal-Wallis test, Welch’s t-test, independent sample’s t-test, and Student’s *t* test. All statistical tests were two-sided. P-values of 0.05 or lower were considered significant.

## Supplementary Material

Supplement 1

## Data Availability

Key datasets used in the analysis will be available upon publication of this manuscript. Other datasets will be available upon request.

## Code availability:

Key customized MATLAB code used in the analysis will be available upon publication of this manuscript.
