# Supplementary material for: A cortical–hippocampal communication undergoes rebalancing after new learning
Source: bioRxiv. 2025 Mar 29:2025.03.26.645547. Preprint. [Version 1] doi: 10.1101/2025.03.26.645547 (PMC11974847; doi:10.1101/2025.03.26.645547)
Supplement: Supplement 1 [file NIHPP2025.03.26.645547v1-supplement-1.pdf]

bioRxiv preprint doi: https://doi.org/10.1101/2025.03.26.645547; this version posted March 29, 2025. The copyright holder for this preprint (which was not certified by peer review) is the author/funder, who has granted bioRxiv a license to display the preprint in perpetuity. It is made available under aCC-BY 4.0 International license.

Supplementary Figures

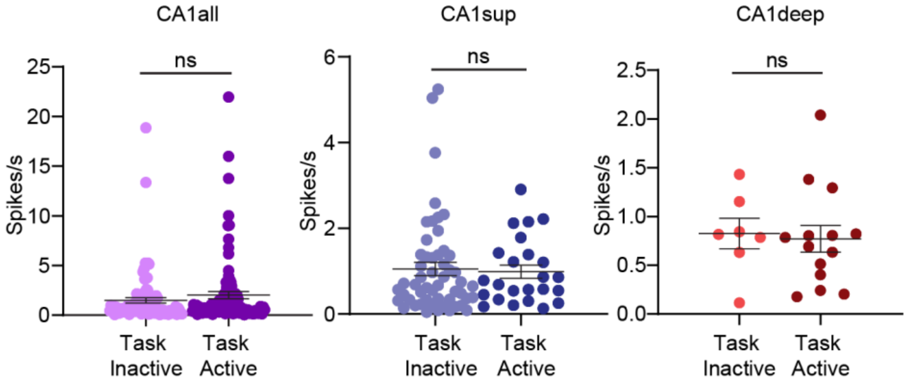

Supplemental Figure 1. Task active and inactive neurons baseline firing rate comparisons. a, Spikes per second calculated during the first ~2-hour pre-training sleep window across cell types. Left, spikes per second comparison across all recorded CA1 neurons including interneurons (n=190). Mann Whitney-U two sided tests revealed no significant between task active (N=95) or inactive (N=95) for all neurons, *P*=.408. Middle, Spikes per second comparison across CA1sup (n=77). Mann Whitney-U two sided tests revealed no significant between task active (N=24) or inactive (N=77) for CA1sup neurons, *P*=.652. Right, Spikes per second comparison across CA1deep (n=21). Independent samples two sided t-tests revealed no significant between task active (N=14) or inactive (N=7) for CA1deep neurons, *P*=.811.

bioRxiv preprint doi: https://doi.org/10.1101/2025.03.26.645547; this version posted March 29, 2025. The copyright holder for this preprint (which was not certified by peer review) is the author/funder, who has granted bioRxiv a license to display the preprint in perpetuity. It is made available under aCC-BY 4.0 International license.

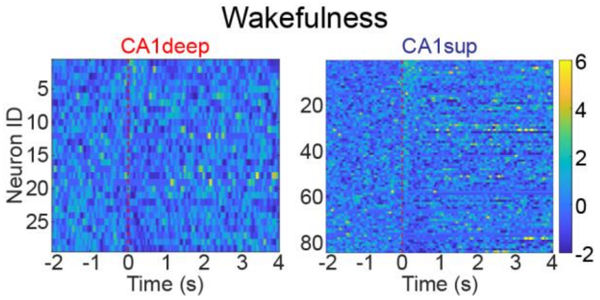

Supplemental Figure 2. CA1deep and CA1sup response to ACC stimulation during wakefulness. Heatmaps for pyramidal neuron spiking (bin 20ms) during wakefulness ACC stimulations.

bioRxiv preprint doi: https://doi.org/10.1101/2025.03.26.645547; this version posted March 29, 2025. The copyright holder for this preprint (which was not certified by peer review) is the author/funder, who has granted bioRxiv a license to display the preprint in perpetuity. It is made available under aCC-BY 4.0 International license.

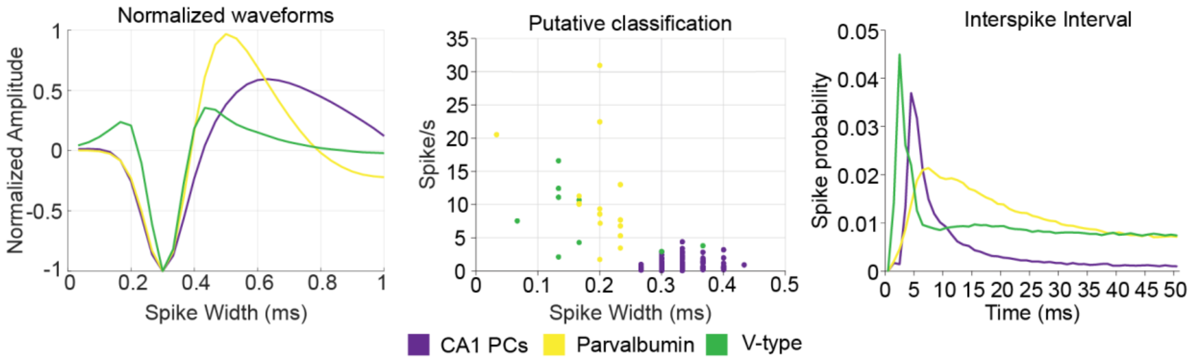

Supplemental Figure 3. Putative Classifications of CA1 neurons. a, Normalized waveform average across all recorded neurons for each neuron subtype in stimulation studies (PCs N=112, PVs n=15, V-type N=9). **b,** Putative classifications of neurons based on neuron firing rate and spike width. **c,** Interspike interval for each neuron subtype. Note, all analyses were performed during sleep portions of recordings.

bioRxiv preprint doi: https://doi.org/10.1101/2025.03.26.645547; this version posted March 29, 2025. The copyright holder for this preprint (which was not certified by peer review) is the author/funder, who has granted bioRxiv a license to display the preprint in perpetuity. It is made available under aCC-BY 4.0 International license.

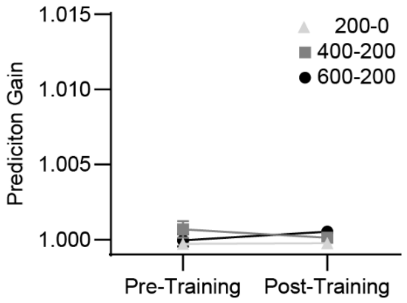

Supplemental Figure 4. Alternate ACC time windows for shuffled data. Shuffled data across three different time windows. There were no differences across any time window for the shuffled data (Kruskal-Wallis' test, p=.145)

bioRxiv preprint doi: https://doi.org/10.1101/2025.03.26.645547; this version posted March 29, 2025. The copyright holder for this preprint (which was not certified by peer review) is the author/funder, who has granted bioRxiv a license to display the preprint in perpetuity. It is made available under aCC-BY 4.0 International license.

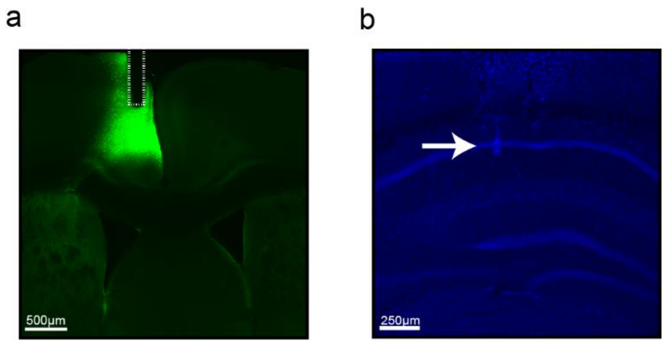

Supplemental Figure 5. Representative histology for optogenetic experiments. a, Optic fiber placement and ChR2 expression. **b,** Tetrode placement for CA1, arrow indicates location of tetrode tip.
